# Extracellular Vesicles from Different Sources of Mesenchymal Stromal Cells Have Distinct Effects on Lung and Distal Organs in Experimental Sepsis

**DOI:** 10.3390/ijms24098234

**Published:** 2023-05-04

**Authors:** Natália G. Blanco, Natália M. Machado, Ligia L. Castro, Mariana A. Antunes, Christina M. Takiya, Monique R. O. Trugilho, Luana R. Silva, Adriana F. Paes Leme, Romênia R. Domingues, Bianca A. Pauletti, Beatriz T. Miranda, Johnatas D. Silva, Claudia C. dos Santos, Pedro L. Silva, Patricia R. M. Rocco, Fernanda F. Cruz

**Affiliations:** 1 Laboratory of Pulmonary Investigation, Carlos Chagas Filho Institute of Biophysics, Federal University of Rio de Janeiro, Rio de Janeiro 21941-902, RJ, Brazil; nataliagoesblanco@gmail.com (N.G.B.);; 2 National Institute of Science and Technology for Regenerative Medicine, Rio de Janeiro 21941-902, RJ, Brazil; 3 Laboratory of Immunopathology, Carlos Chagas Filho Institute of Biophysics, Federal University of Rio de Janeiro, Rio de Janeiro 21941-902, RJ, Brazil; 4 Toxinology Laboratory, Center for Technological Development Health, Oswaldo Cruz Foundation (FIOCRUZ), Rio de Janeiro 21040-900, RJ, Brazil; 5 Mass Spectrometry Laboratory, Brazilian Bioscience National Laboratory (LNBio), Brazilian Center for Research in Energy and Materials, Campinas 13083-970, SP, Brazil; 6 Laboratory of Cellular and Molecular Cardiology, Carlos Chagas Filho Institute of Biophysics, Federal University of Rio de Janeiro, Rio de Janeiro 21941-902, RJ, Brazil; 7 The Keenan Research Centre for Biomedical Science, St. Michael’s Hospital, Unity Health Toronto, 209 Victoria Street, Toronto, ON M5B 1T8, Canada; 8 Institute of Medical Sciences, Temerty Faculty of Medicine, University of Toronto, 1 King’s College Circle, Toronto, ON M5S 1A8, Canada; 9 Department of Physiology, Faculty of Medicine, University of Toronto, 1 King’s College Circle, Toronto, ON M5S 1A8, Canada

**Keywords:** sepsis, extracellular vesicles (EVs), mesenchymal stromal cells (MSCs), animal model, proteomics, immunomodulation

## Abstract

The effects of the administration of mesenchymal stromal cells (MSC) may vary according to the source. We hypothesized that MSC-derived extracellular vesicles (EVs) obtained from bone marrow (BM), adipose (AD), or lung (L) tissues may also lead to different effects in sepsis. We profiled the proteome from EVs as a first step toward understanding their mechanisms of action. Polymicrobial sepsis was induced in C57BL/6 mice by cecal ligation and puncture (SEPSIS) and SHAM (control) animals only underwent laparotomy. Twenty-four hours after surgery, animals in the SEPSIS group were randomized to receive saline or 3 × 10^6^ MSC-derived EVs from BM, AD, or L. The diffuse alveolar damage was decreased with EVs from all three sources. In kidneys, BM-, AD-, and L-EVs reduced edema and expression of interleukin-18. Kidney injury molecule-1 expression decreased only in BM- and L-EVs groups. In the liver, only BM-EVs reduced congestion and cell infiltration. The size and number of EVs from different sources were not different, but the proteome of the EVs differed. BM-EVs were enriched for anti-inflammatory proteins compared with AD-EVs and L-EVs. In conclusion, BM-EVs were associated with less organ damage compared with the other sources of EVs, which may be related to differences detected in their proteome.

## 1. Introduction

Sepsis is one of the leading causes of death worldwide and mortality remains high [1,2,3,4]. Life-threatening organ dysfunction is caused by a dysregulated host response to infection. Infectious agents trigger an immune imbalance, leading to excessive inflammatory response, which causes severe tissue damage and organ failure in the early phase and immunosuppression in the late phase [5].

Mesenchymal stromal cells (MSCs) are known to have immunomodulatory, regenerative, and antimicrobial properties [6,7,8], which have been shown to significantly mitigate immune imbalance, morbidity, and the survival rate in preclinical studies of sepsis [9]. MSCs can act through direct cell-to-cell contact and the release of soluble mediators and extracellular vesicles (EVs) [10,11]. MSCs have already been tested in a phase 1 clinical trial and proved to be safe [12]. In parallel, studies have shown that higher doses of MSCs can reduce the severity of sepsis [13]; however, this may lead to pulmonary embolism [14]. EVs derived from MSCs may be an alternative therapeutic cell-free option [15] because they may prevent this adverse event and maintain adequate hemodynamic stability while preserving the beneficial effects of MSCs [15].

MSCs can be extracted from different sources, such as bone marrow (BM), adipose (AD) and lung (L) tissues, umbilical cord, and menstrual fluid [16]. Although these MSCs are defined according to the International Society of Cell Therapy (ISCT) criteria, MSCs obtained from different sources have different baseline characteristics, which may result in varying preclinical and clinical effects [17,18,19,20]. The proteome of EVs may have different therapeutic biological effects according to the MSC environment [21].

We hypothesized that, in cecal ligation and puncture (CLP)-induced sepsis, MSC-EVs derived from BM, as well as AD and L tissues obtained from the same donors, may lead to diverse effects on the histology and biomarkers associated with lung, kidney, and liver damage. Moreover, in this study, we performed a proteomic analysis of different MSC-derived EVs as a first step toward understanding the possible mechanisms of action of EVs in sepsis, and their differing effects according to the source of the MSCs.

## 2. Results

### 2.1. Characterization of Extracelullar Vesicles (EVs)

**Figure 1:**
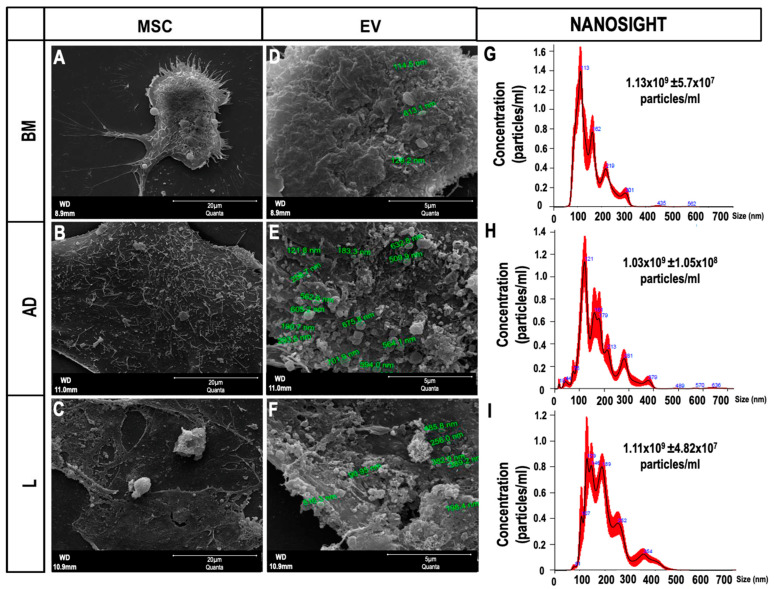
Characterization of extracellular vesicles (EVs). (**A**–**C**) Scanning electron microscopy of mesenchymal stromal cells (MSCs) derived from (**A**) bone marrow (BM), (**B**) adipose tissue (AD), and (**C**) lung tissue (L) 12 h after serum deprivation, showing EVs on the cell surface (Scale bar—20 μm). (**D**–**F**) Higher magnification of (**A**–**C**) showing the EVs (Scale bar—5 μm). (**G**–**I**) Representative graphs obtained by nanoparticle tracking analysis showing the concentration of EVs and two populations of EVs (small and medium/large) obtained from (**G**) BM, (**H**) AD, and (**I**) L.

The ultrastructural profile obtained by scanning electron microscopy of MSCs and EVs derived from the three sources of MSCs is shown in Figure 1A–F. Nanoparticle tracking analysis showed two populations of EVs obtained from the three sources of MSCs, one of lower intensity and small size (<200 nm, classified by the International Society for Extracellular Vesicles as small EVs), and another with higher intensity and average size (>200 nm, classified as medium/large EVs) [15]. The mean molecular diameter of BM-EVs was smaller (153.0 ± 2.1 nm) than that of AD-EVs (176.0 ± 3.6 nm) and L-EVs, which were the largest (198.5 ± 3.1 nm). Similar concentrations of EVs were observed after ultracentrifugation of the supernatants obtained from MSCs derived from BM (1.1 × 10^9^ ± 5.7 × 10^7^ particles/mL), AD (1.03 × 10^9^ ± 1.05 × 10^8^ particles/mL), and L (1.1 × 10^9^ ± 4.82 × 10^7^ particles/mL) (Figure 1G–I).

### 2.2. Proteomics

#### 2.2.1. Protein in Extracellular Vesicles

**Figure 2:**
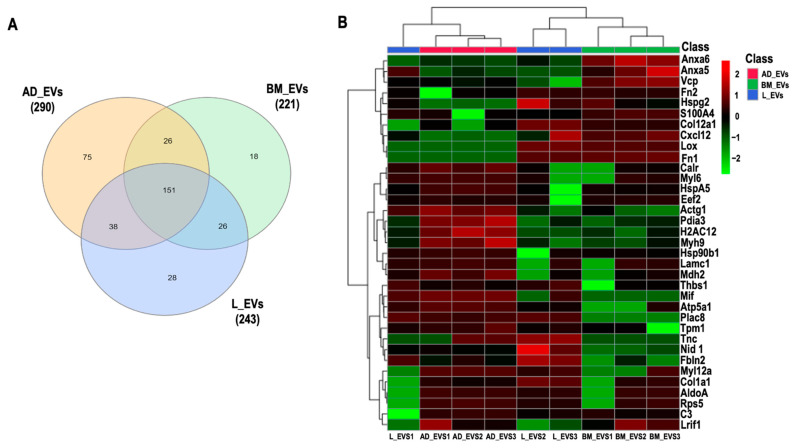
Proteomic analysis. (**A**) Venn diagram of common and “exclusive” proteins identified for EVs derived from bone marrow (BM), adipose (AD), and lung (L) extracellular vesicles (EVs). (**B**) Unsupervised hierarchical clustering analysis of differentially abundant proteins (log_2_LFQ intensity values) from BM-, AD-, and L-derived EVs using Euclidian distance measure and Ward algorithm clustering. Actin cytoplasmic 2 (Actg1), fructose bisphosphate aldolase A (AldoA), annexin A5 (Anxa5), annexin A6 (Anxa6), ATP synthase subunit alpha, mitochondrial (Atp5a1), complement C3 (C3), calreticulin (Calr), collagen alpha 1(XII) chain (Col12a1), collagen alpha 1 (Col1a1), stromal cell-derived factor 1 (Cxcl12), elongation factor 2 (Eef2), fibulin 2 (Fbln2), fibronectin isoform 1 (Fn1), fibronectin isoform 2 (Fn2), histone H2A type 1H (H2AC12), endoplasmin (Hsp90b1), 78 kDa glucose-regulated protein (HspA5), basement membrane-specific heparan sulfate proteoglycan core protein (Hspg2), laminin subunit gamma 1 (Lamc1), protein lysine 6 oxidase (Lox), ligand-dependent nuclear receptor interacting factor 1 (Lrif1), malate dehydrogenase 2 (Mdh2), macrophage migration inhibitory factor (Mif), myosin 9 (Myh9), myosin light chain 12A (Myl12a), myosin light polypeptide 6 (Myl6), nidogen 1 (Nid 1), protein disulfide isomerase A3 (Pdia3), placenta specific 8 (Plac8), ribosomal protein S5 (Rps5), S100 calcium-binding protein A4 (S100A4), thrombospondin 1 (Thbs1), tenascin (Tnc), tropomyosin alpha-1 chain (Tpm1), transitional endoplasmic reticulum ATPase (Vcp).

A total of 362 proteins (Figure 2A) were identified and quantified (filters applied: reverse and only identified by site) in triplicates of MSC-derived EVs from BM, AD, and L; 44 proteins were considered to be statistically abundant (ANOVA, *p* < 0.05). After filtering the contaminants, the clustering of the remaining 35 proteins present in significant abundance was visualized using hierarchical clustering analysis (Figure 2B).

**Figure 3:**
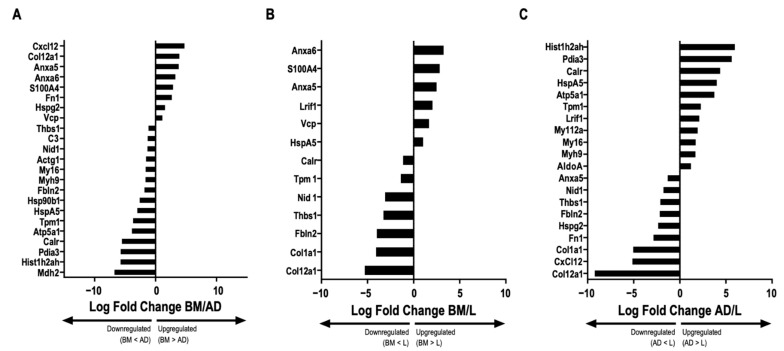
Comparative abundance of proteins in extracellular vesicles derived from different sources. Graphs represent the log_2_ ratio of the LFQ intensity comparing the abundance of proteins in extracellular vesicles (EVs) derived from each source: bone marrow (BM)/adipose tissue (AD), BM/lung tissue (L), and AD/L. (**A**) BM/AD, indicating upregulation and downregulation between the groups (ANOVA followed by Tukey’s honest significant difference [HSD], *p <* 0.05). (**B**) BM/L, indicating upregulation and downregulation between the groups (ANOVA followed by Tukey’s HSD, *p* < 0.05). (**C**) AD/L, indicating upregulation and downregulation between the groups (ANOVA followed by Tukey’s HSD, *p* < 0.05). Actin cytoplasmic 2 (Actg1), fructose bisphosphate aldolase A (AldoA), annexin A5 (Anxa5), annexin A6 (Anxa6), ATP synthase subunit alpha, mitochondrial (Atp5a1), complement C3 (C3), calreticulin (Calr), collagen alpha 1(XII) chain (Col12a1), collagen alpha 1 (Col1a1), stromal cell-derived factor 1 (Cxcl12), elongation factor 2 (Eef2), fibulin 2 (Fbln2), fibronectin isoform 1 (Fn1), fibronectin isoform 2 (Fn2), histone H2A type 1H (H2AC12), histone H2A type 1H (Hist1h2ah), endoplasmin (Hsp90b1), 78 kDa glucose-regulated protein (HspA5), basement membrane-specific heparan sulfate proteoglycan core protein (Hspg2), laminin subunit gamma 1 (Lamc1), protein lysine 6 oxidase (Lox), ligand-dependent nuclear receptor interacting factor 1 (Lrif1), malate dehydrogenase 2 (Mdh2), macrophage migration inhibitory factor (Mif), myosin 9 (Myh9), myosin light chain 12A (Myl12a), myosin light polypeptide 6 (Myl6), nidogen 1 (Nid 1), protein disulfide isomerase A3 (Pdia3), placenta specific 8 (Plac8), ribosomal protein S5 (Rps5), S100 calcium-binding protein A4 (S100A4), thrombospondin 1 (Thbs1), tenascin (Tnc), tropomyosin alpha-1 chain (Tpm1), transitional endoplasmic reticulum ATPase (Vcp).

The full protein names are listed in Table S2. No protein exclusive to one source within the EVs was identified. Cluster of differentiation (CD)81, an exosome surface marker [6], and CD44, an MSC surface marker [6], were identified in all EVs derived from the three cell types. Pairwise comparisons were used to identify differential protein abundance in MSC-derived EVs from BM, AD, and L (BM/AD, BM/L, and AD/L) (Figure 3A–C). We observed that annexin A5 (Anxa5), S100 calcium-binding protein A4 (S100A4), and transitional endoplasmic reticulum ATPase (Vcp) were upregulated in MSC-derived EVs from BM compared with the other sources, and calreticulin (Calr), tropomyosin alpha-1 chain (Tpm1), Nidogen (Nid 1), thrombospondin 1 (Thbs1), and fibulin 2 (Fbln2) were downregulated. MSC-derived EVs from AD were enriched for histone H2A type (Hist1h2ah), protein disulfide isomerase A3 (Pdia3), Calr, 78 kDa glucose-regulated protein (HspA5), ATP synthase subunit alpha, mitochondrial (Atp5a1), tropomyosin alpha-1 chain (Tpm1), myosin light polypeptide 6 (My16), and myosin 9 (Myh9); and Anxa5, fibronectin isoform 1 (Fn1), heparan sulfate proteoglycan core protein (Hspg2), stromal cell-derived factor 1 (Cxcl12), and collagen alpha 1 (XII) chain (Col12a1) were downregulated compared with MSC-derived EVs from BM and L. MSC-derived EVs from L-derived MSCs expressed less Thbs1, Fbln2, collagen alpha 1 (Col1a1), and Col12a1 compared with those released from BM and AD.

#### 2.2.2. Predicted Pathway Enrichment in Bone Marrow (BM)-, Adipose Tissue (AD)-, and Lung (L)-Derived EVs

Based on the list of proteins up- and downregulated in each group, we performed pathway enrichment analysis with WikiPathways. Related pathways are described in Table S3.

MSC-derived EVs from BM were predicted to lead to activation of interleukin (IL)-9 signaling through Vcp, prostaglandin synthesis, and regulation through Anxa5 pathways, as well as downregulation of the inflammatory response, transforming growth factor (TGF)-β signaling, P53 signaling, and focal adhesion pathways, which are all associated with Thbs1.

Upregulation of oxidative phosphorylation and electron transport chain pathways through Atp5a1 and downregulation of the inflammatory response, regulation of the actin cytoskeleton and focal adhesion pathways—which, in this case, are related to Fn1—and the downregulation of prostaglandin synthesis and regulation pathways related to Anxa5 must be mentioned with regard to MSC-derived EVs from AD.

### 2.3. Survival Rate

Twenty-four hours after therapy, septic animals treated with BM-EVs, AD-EVs, L-EVs, and SAL, compared with the SHAM group, presented survival of 89, 60, 88, and 77%, respectively. No animals died in the SHAM group (100% survival rate).

### 2.4. Lung Histology and Molecular Biology

**Figure 4:**
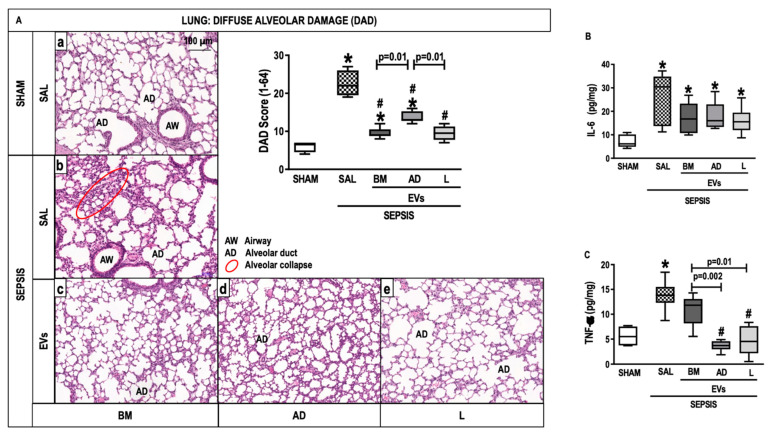
Lung Histology and Molecular Biology. (**A**) Representative photomicrographs of lung stained with hematoxylin and eosin from the groups: (a) SHAM, (b) SEPSIS-SAL, (c) SEPSIS-Extracellular vesicles (EVs)-bone marrow (BM), (d) SEPSIS-EVs-adipose tissue (AD), and (e) SEPSIS-EVs-lung tissue (L). Bars represent 100 µm. Diffuse alveolar damage (DAD) score, Airway (AD), Alveolar duct (AD), Alveolar collapse (red circle). (**B**,**C**), Interleukin (IL)-6 and tumor necrosis factor (TNF)-α levels, respectively. Data are presented as box plots of medians and interquartile ranges with 6 animals in each group. * Significantly different from SHAM (adjusted *p*-value < 0.0125). # Significantly different from SAL (*p* < 0.05).

The cumulative difuse alveolar damage (DAD) score was higher in the SEPSIS-SAL group than in the SHAM group (*p* < 0.0125) (Figure 4A). All septic animals treated with EVs had a reduced DAD score compared with those treated with saline (Figure 4A). Levels of IL-6 and tumor necrosis factor (TNF)-α were higher in the SEPSIS-SAL group compared with the SHAM (*p* < 0.0125). IL-6 expression in lung tissue did not significantly decrease after EV therapy (Figure 4B), but the level of TNF-α expression reduced in all treated groups with EVs (Figure 4C).

### 2.5. Kidney Histology and Molecular Biology

**Figure 5:**
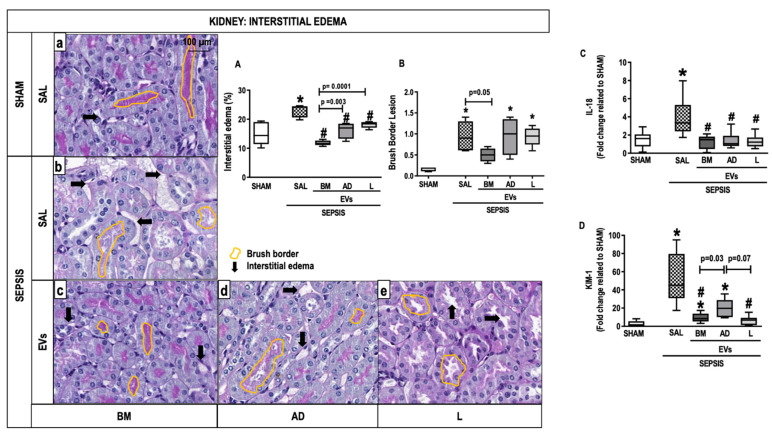
Kidney Histology and Molecular Biology. Representative photomicrographs of kidney stained with periodic acid Schiff- from the groups: (a) SHAM, (b) SEPSIS-SAL, (c) SEPSIS-Extracellular vesicles (EVs)-bone marrow (BM), (d) SEPSIS-EVs-adipose tissue (AD), and (e) SEPSIS-EVs-lung tissue (L). Bars represent 100 µm. Interstitial edema (**A**) and brush border (**B**) lesions were quantified. Arrows, interstitial edema; yellow outlines, brush border. Gene expressions of interleukin (IL)-18 (**C**) and kidney injury molecule (KIM)-1 were quantified by RT-PCR. Data are presented as box plots of medians and interquartile ranges with 6 animals in each group. * Significantly different from SHAM (adjusted *p*-value < 0.0125). # Significantly different from SAL (*p* < 0.05).

Animals from the SEPSIS-SAL group showed interstitial edema as well as brush border lesions compared with the SHAM group (Figure 5A). The three sources of EVs, especially BM-EVs, decreased interstitial edema compared with SEPSIS-SAL animals (Figure 5A). The brush border lesions were reduced after BM-EVs (Figure 5B). Expression of IL-18 and kidney injury molecule (KIM)-1 was higher in the SEPSIS-SAL group compared with the SHAM group. The administration of EVs derived from all three sources reduced IL-18, whereas only BM-EVs and L-EVs were able to decrease the expression of KIM-1 compared with SAL animals (Figure 5C,D).

### 2.6. Liver Histology and Molecular Biology

**Figure 6:**
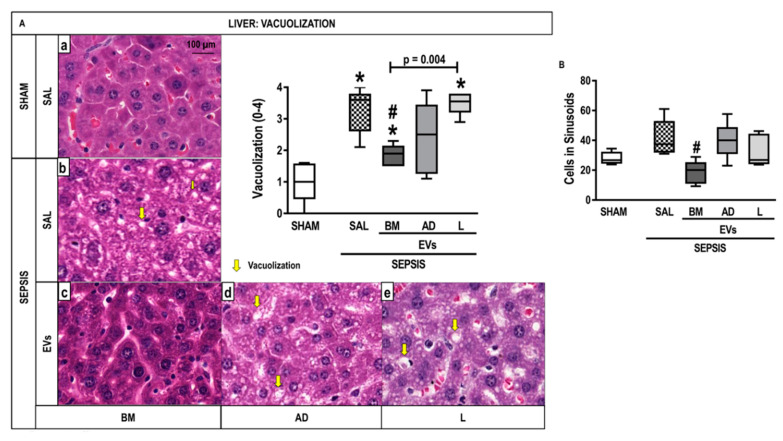
Liver Histology and Molecular Biology. (**A**) Representative photomicrographs of liver stained with hematoxylin and eosin from the groups: (a) SHAM, (b) SEPSIS-SAL, (c) SEPSIS-Extracellular Vesicles (EVs)-bone marrow (BM), (d) SEPSIS-EV-adipose tissue (AD), and (e) SEPSIS-EV-lung tissue (L). Bars represent 100 µm. Note the presence of liver vacuolization (steatosis and hydropic degeneration) (yellow arrows). The number of cells in hepatic sinusoids (inflammation) (**B**) was also quantified. Data are presented as box plots of medians and interquartile ranges with 6 animals in each group. * Significantly different from SHAM (adjusted *p*-value < 0.0125). # Significantly different from SAL (*p* < 0.05).

The score for nuclear vacuolization in hepatocytes was higher in the SEPSIS-SAL group compared with the SHAM group but was attenuated after treatment with BM-EVs (Figure 6A). BM-EV therapy reduced the number of cells in sinusoids compared with SEPSIS-SAL animals (Figure 6B). No significant differences were observed in the expression of IL-6, IL-10, and programmed cell death protein (PD)-1 in liver tissue among the groups (Figure S1).

## 3. Discussion

In this study, MSC-derived EVs from BM, as well as AD, and L tissues had different histologic and biological effects on the injury profile of lung, kidney, and liver in experimental CLP-induced sepsis. In the lung, EVs from BM, AD, and L were able to reduce the DAD score and TNF-α expression in lung parenchyma. In the kidney, EVs reduced interstitial edema regardless of the source of MSCs. Acute kidney injury biomarkers, such as IL-18 and KIM-1, decreased after treatment with EVs from BM and L. In the liver, BM-EVs decreased both the nuclear vacuolization score in hepatocytes and the number of cells in the sinusoids. BM-EVs were associated with less lung, liver, and kidney damage compared with AD-EVs and L-EVs, which led to the hypothesis related to differences in EV proteomic content.

According to the International Society of Cell and Gene Therapy, MSCs are characterized by the ability to adhere to plastic and to differentiate into chondroblasts, adipocytes, and osteoblasts, and by the expression of specific surface markers (CD105, CD73, and CD90) and by the lack of CD45, CD34, and CD14 [22]. These cells can home to inflamed tissues through a chemoattractor effect by signaling molecules released from immune and injured cells, and then act in organ repair by delivering trophic and growth factors, such as TGF-β1. They also have anti-inflammatory, antimicrobial, antiapoptotic, and antioxidative effects, which are crucial in the early phase of sepsis therapy [7]. MSCs increased M2 macrophage polarization, reversed the increase in proinflammatory cytokines, and increased Treg function [7,23,24]. BM is still the most studied source of MSCs, but new sources are emerging, such as AD and L tissues, umbilical cord, menstrual fluid, and others. The phenotypic, functional, and immunologic properties of MSCs depend on their source [17,25].

EVs are secreted by several types of cells and can be classified by their physical characteristics, including size (small, <200 nm; medium/large, >200 nm) [15]. They contain proteins, lipids, DNA, mRNA, miRNA, and cell organelles, with profiles that are similar to their cells of origin [6,16], which change according to the MSC source, even when derived from the same donor [26].

The well-known CLP model was chosen to induce experimental sepsis because it closely mimics some aspects of sepsis pathophysiology in humans [27,28]. According to previous studies, 6 h after CLP, mice present increased systemic inflammation [29], and 24 h after surgery, functional and morphologic changes in the lung, kidney, and liver are observed [29]. For translational reasons, antibiotics and fluids were administered after induction of CLP.

We selected ultracentrifugation at 100,000× *g* to isolate EVs, a lower speed than previously reported [30]. According to the most recent consensus from the International Society of Extracellular Vesicles [15], there is no optimal separation method to isolate EVs, and it must be chosen based on the expertise and goal of the scientist. The protocol chosen in this study guarantees intermediate recovery and specificity of EVs, which is adequate to simulate one of the mechanisms of action of MSCs: the release of EVs into circulation. We observed that the EVs obtained with our protocol had the size (between 150 and 200 nm) and morphology (spherical shape) expected according to the consensus [15]. EVs were intravenously injected 24 h after CLP surgery when sepsis was already established [31,32,33].

The trend toward reduced survival after AD-EVs compared with SHAM may be associated with a lower content of Fn1 (a protein that interacts with fibrin during the coagulation process) in AD-EVs compared with other EV sources. Lower Fn1 in plasma has been previously described as a prognostic marker for human sepsis and may be related to the host response to infection [34,35,36,37]. AD-EVs also showed less Anxa5, an anticoagulant protein that has a protective effect on sepsis coagulopathy [38], and activated ATP-related pathways, which may be associated with the production of massive reactive oxygen species and proinflammatory pathways. Further studies are required to confirm the role of the proteomic content present in AD-EVs.

Regarding the effects of EVs on the lungs, the three sources were able to reduce the cumulative DAD score. BM-EVs reduced kidney interstitial edema and the presence of early biomarkers of proximal tubule injury, such as KIM-1 and IL-18 [39], to a greater extent than AD-EVs and L-EVs. BM was the only source of EVs able to reduce liver damage, as shown by quantification of the nuclear vacuolization of hepatocytes and by cell infiltration on sinusoids, even though no positive effects were observed on the expression of IL-6, IL-10, and PD1. Comparing the proteomic content of BM-EVs with AD-EVs and L-EVs, we observed a higher concentration of Vcp, a protein that stimulates the IL-9 signaling pathway, as described in Table S3. An increase in IL-9 acts to protect septic mice via a mechanism involving the modulation of proinflammatory and anti-inflammatory mediators, which may improve the survival rate in sepsis [40]. In addition, it was demonstrated that BM-EVs have more Anxa5, an anticoagulant factor that protects against multiple organ dysfunction in sepsis.

EVs derived from MSCs might be an alternative to cell therapy to reduce the clinical adverse events associated with cell administration, such as thromboembolic events. According to our results, the effects of EV therapy in the current model of experimental sepsis depend on the cell source and the target organ. Therefore, it would be beneficial to provide therapy for humans with EVs that have the best performance on the most damaged organ in each disease, orchestrating an optimized and less expensive approach. More studies are required to comprehend the best EV source for each injured organ and pathology.

## 4. Materials and Methods

### 4.1. Study Approval

This study was approved by the Ethics Committee of the Health Sciences Center (CEUA-025/17) at the Federal University of Rio de Janeiro. All animals received proper care according to the instructions formulated by the National Society for Medical Research and by the U.S. National Academy of Sciences. The present study followed the ARRIVE guidelines for reporting of animal research [41]. Conventional animals were housed at a controlled temperature (23 °C) and in a controlled light–dark cycle (12–12 h) with free access to water and food.

### 4.2. Extraction and Characterization of Mesenchymal Stromal Cells and Extracellular Vesicles

Twelve healthy male C57BL/6 mice (20–25 g, 6–8 weeks old) were euthanized with intravenous ketamine (25 mg/kg; Cristália, Itapira, SP, Brazil) and xylazine (2 mg/kg; Xilazin, Syntec, Barueri, SP, Brazil) and used as donors. MSCs from BM, AD (epididy-mal fat pad), and L were obtained and harvested as previously described [19]. Four different individuals were pooled for the isolation of MSCs. Three replicates were used in the analysis.

Stress induction of BM-, AD-, and L-MSCs was achieved by full serum depletion in the culture medium to induce a massive release of EVs [30]. After 12 h, the conditioned medium was collected and centrifuged at 2000× *g* for 20 min at 4 °C to remove cellular debris, followed by ultracentrifugation (100,000× *g*) for 1 h at 4 °C. The precipitate was collected and suspended in 0.9% saline solution for immediate use or suspended in 100 µL of 50 mM NH4HCO3 with 0.2% RapiGest SF (Waters Corporation, Milford, MA, USA) and frozen (−80 °C) for proteomics analysis. The hydrodynamic diameter and concentration of EVs were evaluated. All samples were diluted 1:10 in 1x sterile filtered phosphate-buffered saline (PBS) to a final volume of 1 mL. According to the manufacturer’s software manual (NanoSight NS300 User Manual, MAN0541-02-EN, 2018; NanoSight, Salisbury, Wiltshire, UK), five 60-s videos were captured under the following conditions: temperature, 25 °C; syringe speed, 25 µL/s; camera level, 13; screen gain, 1.0. After capture, the videos were analyzed by the in-built NanoSight software (NTA 3.4 build 3.4.4, Marvern Panalytical, Malvern, Worcestershire, UK) with a detection threshold of 7. The hardware included an embedded laser at 532 nm and a sCMOS camera [42].

### 4.3. Proteomics

#### 4.3.1. Sample Preparation

The concentration of the protein content was estimated by the absorbance reading at 280 nm (NanoDrop 2000, Thermo Fisher Scientific, Wilmington, DE, USA). The protein content of approximately 30 µg of EVs was reduced using dithiothreitol (10 mM final concentration, 3 h at 37 °C) and alkylated with iodoacetamide (25 mM final concentration, 30 min in the dark at room temperature). Extracted proteins were incubated with trypsin (Promega, Madison, WI, USA) 1:50 (*w*/*w*) for ~20 h at 37 °C and 45 min at 56 °C thermoblock (Eppendorf, Hamburg, Hamburg, Germany). The reaction was stopped by adding trifluoroacetic acid to a final concentration of 1% (*v*/*v*). The digested peptide mixture was desalted in reverse-phase homemade microcolumns with POROS R2 resin (Applied Biosystems, Waltham, MA, USA). Samples were dried completely in a vacuum centrifuge (Savant Speed Vac Plus SC210A; Thermo Fisher Scientific, Waltham, MA, USA) and resuspended with 1% formic acid. The concentration of the peptide mixture was estimated by the absorbance reading at 280 nm (NanoDrop 2000, Thermo Fisher Scientific, Wilmington, DE, USA). Samples were stored at −20 °C for MS analysis.

#### 4.3.2. Mass Spectrometry Analysis

The peptide mixture (4.0 µL of EV samples) was analyzed using an LTQ Orbitrap Velos mass spectrometer (Thermo Fisher Scientific, Bremen, Germany) coupled to nanoflow liquid chromatography on an EASY-nLC system (Proxeon Biosystems, Odense, Denmark) with a Proxeon nanoelectrospray ion source. Peptides were subsequently separated in a 290% acetonitrile gradient in 0.1% formic acid using a PicoFrit analytical column (20 cm × 75.5 µm inner diameter; New Objective, Woburn, MA, USA) at a flow rate of 300 nL/min over 212 min (MSC samples) and 80 min (EV samples); a gradient of 35% acetonitrile was reached in 175 min and 40 min, respectively. The nanoelectrospray voltage was set to 2.2 kV, and the source temperature was set to 275 °C. The LTQ Orbitrap Velos was set up in data-dependent acquisition mode. Full scan MS spectra (m/z 300–1600) were acquired in the Orbitrap analyzer after accumulation to a target value of 1 × 10^6^. The resolution in the Orbitrap was set to *r* = 60,000, and the 20 most intense peptide ions (top 20) with charge states ≥2 were sequentially isolated to a target value of 5000 and fragmented in the high-pressure linear ion trap by collision-induced dissociation with a normalized collision energy of 35%. Dynamic exclusion was enabled with an exclusion size list of 500 peptides, an exclusion duration of 60 s, and a repetition count of 1. An activation Q of 0.25 and an activation time of 10 ms were used [43]. The run order was randomized and blocked using the open-source statistical programming language R (R Foundation for Statistical Computing, Vienna, Austria).

#### 4.3.3. Proteomic Data Analysis

Raw data were processed using MaxQuant v1.5.3.8 software (Max Planck Institute of Biochemistry, Martinsried, Germany) [44], and MS/MS spectra were searched against the mouse UniProt database (released December 2020, 63,724 sequences and 28,586,808 residues) using the Andromeda search engine [45]. As search parameters, a tolerance of 10 ppm was considered for precursor ions (MS search) and 1 Da for fragment ions (MS/MS search), with a maximum of two missed cleavages. Carbamidomethylation of cysteine was considered a fixed modification, and oxidation of methionine and protein N-terminal acetylation were considered variable modifications. A maximum false discovery rate of 1% was set for both protein and peptide identification. Protein quantification was performed using the LFQ algorithm implemented in MaxQuant software, with a minimum ratio count of 1 and a window of 2 min for matching between runs. Statistical analysis was performed with Perseus v.1.6.5.0 software (Max Planck Institute of Biochemistry, Martinsried, Germany) [46], which is available in the MaxQuant package. Protein entries identified were processed, excluding reverse sequences and those identified “only by site” entries. Contaminants were not removed from the dataset. For all statistical comparisons, a *p*-value ≤ 0.05 was used to define significance (analysis of variance (ANOVA)).

#### 4.3.4. Proteomic Data Interpretation

For data visualization, heatmaps with z-score values of log_2_LFQ intensities were built with Metaboanalyst v 5.0 software (McGill Computational and Data Systems Initiative, Montreal, Quebec, Canada) using Euclidian distance measure and Ward algorithm clustering. Heatmaps were built from the proteins identified in EVs with a *p*-value < 0.05, excluding potential contaminants. The most abundant proteins were identified in each population of EVs in comparison with the other two populations (only those with a *p*-value < 0.05 were included) and were decoded using the String Platform (https://string-db.org/, accessed on 19 March 2021) for murine analysis. The significant proteins were enriched through the *Enricher* Platform (https://maayanlab.cloud/Enrichr/, accessed on 19 March 2021), considering indexed pathways on WikiPathways 2019 Mouse.

### 4.4. Scanning Electron Microscopy

Twelve hours after culturing BM-, AD-, and L-MSCs in serum-free medium, cells and extracted EVs were fixed in 2.5% glutaraldehyde in 0.1 M sodium cacodylate buffer (pH 7.2) for 2 h and washed twice with cacodylate buffer. Post-fixation with OsO_4_ and FeCNK solution (1:1) for 45 min was immediately performed, followed by dehydration in a graded ethanol series for 10 min at each concentration (30, 50, 70, 90, 100%, the latter three times). After critical point drying, the coverslips were analyzed and images were acquired in an FEI QUANTA 250 scanning electron microscope (FEI, Hillsboro, OR, USA). The coverslip with BM-, AD-, and L-MSCs not subjected to serum depletion stress was used as a control.

### 4.5. Animal Preparation and Experimental Protocol

**Figure 7:**
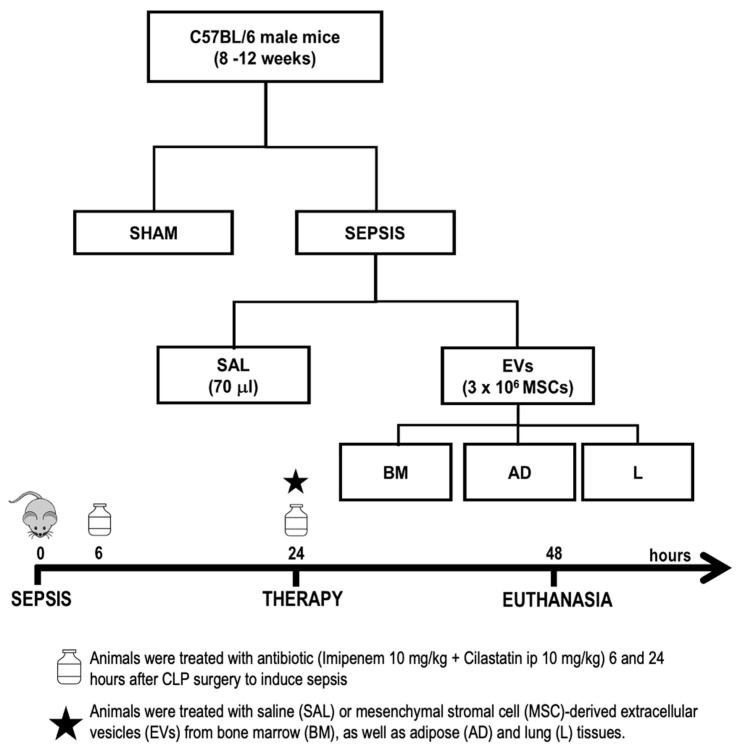
Experimental design. Male mice were anesthetized, and sepsis was induced by cecal ligation and puncture (CLP). Sham animals underwent the same surgical procedure without CLP. Septic mice intraperitoneally received imipenem + cilastatin 6 and 24 h after induction of sepsis. After 24 h, they were intravenously treated with saline (SAL, 70 mL) or EVs (3 × 10^6^ mesenchymal stromal cells (MSCs)) derived from bone marrow (BM), adipose (AD), and lung (L) tissues. Forty-eight hours after induction of sepsis, the animals were euthanized and the lungs, liver, and kidneys were removed for further analysis.

All experimental assessments were performed in a blind fashion. Thirty male C57BL/6 mice (25–30 g, 8–12 weeks old) were allocated into two main groups: sham (SHAM) (*n* = 6) and experimental sepsis (SEPSIS) (*n* = 24) induced by CLP surgery [47]. Twenty-four hours after induction of sepsis, septic animals were randomly assigned using sealed envelopes to subgroups (*n* = 6/subgroup) and intravenously received the following: (1) sterile saline solution (SAL, 70 μL); (2) MSC-derived EVs from BM (3 × 10^6^, 70 μL); (3) MSC-derived EVs from AD (3 × 10^6^, 70 μL); and (4) MSC-derived EVs from L (3 × 10^6^, 70 μL) (Figure 7). Twenty-four hours after treatment, the lungs, liver, and kidneys were removed to evaluate the histology and molecular biology.

### 4.6. Survival Rate

Survival studies were performed on an additional 50 male C57BL/6 mice (*n* = 10/group, 25–30 g, 8–12 weeks old). They were allocated into two main groups, SHAM and SEPSIS, and then into four additional groups (SAL, BM, AD, and L). Animals were monitored, and deaths were evaluated daily during the first 48 h.

### 4.7. Histology

The cumulative diffuse alveolar damage (DAD) score was quantified [48]. Dear’s adapted score [49] was used to assess the degree of damage in the liver. Moreover, inflammation in the liver was assessed by counting the total number of polymorphonuclear cells in sinusoids. The percentage of interstitial edema and brush border lesions was quantified in the kidney. Histologic analysis of the lung, liver, and kidney was performed by three examiners blinded to group assignment (N.G.B., J.D.S., and C.M.T.) (Supplementary Materials and Methods).

### 4.8. Enzyme-Linked Immunosorbent Assay

IL-6 and TNF-α were quantified by enzyme-linked immunosorbent assay (ELISA) in lung tissue homogenate as per the manufacturer’s protocol (Peprotech, Cranbury, NJ, USA) and normalized to the total protein content quantified by Bradford’s reagent (Sigma-Aldrich, St Louis, MO, USA).

### 4.9. Real-Time Reverse Transcription Polymerase Chain Reaction Analysis (RT-PCR)

A quantitative real-time reverse transcription polymerase chain reaction (RT-PCR) was used to measure the expression of selected mediators in the kidney (*IL-18* and *KIM-1*) and liver homogenates (*IL-6*, *IL-10*, and *PD-1*). 36B4 (*acidic ribosomal phosphoprotein P0*) was used as the housekeeping gene. The primers are listed in Table S1.

### 4.10. Statistical Analysis

One-way ANOVA followed by Holm–Šidák multiple comparisons test and Kruskal–Wallis followed by Dunn’s test were used for parametric and nonparametric data, respectively. Parametric data are expressed as means ± standard deviation and nonparametric data as medians (interquartile range). Survival curves were derived by the Kaplan–Meier method and compared by log-rank test. All tests were performed using Prism 8 software (GraphPad Software, La Jolla, CA, USA) and statistical significance was established at a *p*-value < 0.05.

## 5. Conclusions

In this experimental sepsis model, BM-derived EVs seem to be associated with a reduction in lung, liver, and kidney damage compared with AD-EVs and L-EVs, which may be related to differences detected in their proteomic content.

## Data Availability

The mass spectrometry (MS) raw data have been deposited in the ProteomeXchange Consortium via the PRIDE [50] partner repository with the dataset identifier PXD023259.

## Supplementary Materials

The supporting information can be downloaded at: https://www.mdpi.com/article/10.3390/ijms24098234/s1.
