# Supplementary material for: Extracellular Vesicles from Different Sources of Mesenchymal Stromal Cells Have Distinct Effects on Lung and Distal Organs in Experimental Sepsis
Source: Int J Mol Sci. 2023 May 4;24(9):8234. doi: 10.3390/ijms24098234 (PMC10179270; doi:10.3390/ijms24098234)
Supplement: Supplementary file 1 [file ijms-24-08234-s001.zip › ijms-2360157-supplementary.pdf]

Supplementary digital content

Materials and Methods

Animal Preparation and Experimental Protocol

Thirty male C57BL/6 mice (25–30 g, 8–12 weeks old) were allocated to two main groups: sham (SHAM) and experimental sepsis (SEPSIS) induced by cecal ligation and puncture (CLP) surgery[1]. Briefly, animals were anesthetized with intraperitoneal (i.p.) injection of ketamine (25 mg/kg, Cristália, Itapira, SP, Brazil) and xylazine (2 mg/kg; Xilazin, Syntec, Barueri, SP, Brazil) and a midline laparotomy (2-cm incision) was performed. The cecum was carefully isolated and ligated below the ileocecal valve with a 3-0 cotton suture to prevent obstruction. Then, the cecum was punctured once with an 18-gauge needle. and the animals were left to recover from anesthesia[1]. In sham surgery, the abdominal cavity was opened, and the cecum was isolated without ligation or puncture. Postoperative care was similar in both groups and consisted of a subcutaneous injection of tramadol hydrochloride (20 mg/g body weight; Cristália, Itapira, SP, Brazil) in 1 mL of warm (37 °C) normal saline (NaCl 0.9%). All animals subjected to CLP surgery received antibiotics (imipenem/cilastatin, 10 mg/kg, i.p., Cristália, Itapira, SP, Brazil) 6 and 24 h after induction of sepsis, as recommended for preclinical studies assessing potential human therapeutics[2]. The well-being of the mice was constantly monitored based on predetermined clinical findings.[3]

Twenty-four hours after induction of sepsis, animals with sepsis (SEPSIS) were randomly assigned by sealed envelopes to the following subgroups (n = 6/subgroup): (1) sterile saline solution (SAL, 70 μL i.v.); (2) extracellular vesicle (EVs) released from 3×10^6 mesenchymal stromal cells (MSCs) derived from bone marrow (70 μL i.v.); (3) EVs released from 3×10^6 MSCs derived from adipose tissue (70 μL i.v.); and (4) EVs released from 3×10^6 MSCs derived from lung tissue (70 μL i.v.) (Fig. 1). For administration of EVs, animals were anesthetized with sevoflurane (1.0%, Sevorane; Cristália, Itapira, SP, Brazil), supported in a supine position, and the jugular vein was carefully exposed. EVs were instilled i.v. and the neck skin was sutured. The animal was then placed in the prone position on the heating bed and observed until return of normal spontaneous breathing. Twenty-four hours after

treatment, lung, liver, and kidney histology was analyzed, and protein and mRNA levels related to organ damage were evaluated.

Histology

Light Microscopy of Lungs, Liver, and Kidney

Animals were euthanized by sodium thiopental injection (50 mg/kg i.v.; Thiopentax; Cristália, Itapira, SP, Brazil). A laparotomy was performed, and heparin (1000 IU, Cristália, Itapira, SP, Brazil) was injected into the vena cava. The trachea was clamped at end expiration (positive end-expiratory pressure = 2 cmH_2O), and the abdominal aorta and vena cava were sectioned, resulting in a massive hemorrhage that quickly killed the animals. The left lung, a slice of the liver, and the left kidney were then removed, fixed in 3% buffered formaldehyde (Isofar, Duque de Caxias, RJ, Brazil) and embedded in paraffin (EasyPath Diagnósticos, Insaiatuba, SP, Brazil). Sections (4 μm thick) were cut and stained with hematoxylin (Isofar, Duque de Caxias, RJ, Brazil) and eosin (Vetec Química, Duque de Caxias, RJ, Brazil) (lung and liver) or periodic acid-Schiff (Vetec Química, Duque de Caxias, RJ, Brazil) (kidney). Slides were scanned with a Panoramic MIDI II scanner (3DHISTECH, Budapest, Hungary).

Diffuse alveolar damage (DAD) was quantified in scanned photomicrographs at magnifications of ×100, ×200, and ×400, obtained from ten non-overlapping fields of view per animal, using a weighted scoring system as described elsewhere[4]. Briefly, the following histologic features were graded for severity from 0 (no effect) to 4 (maximum severity): (1) edema, (2) alveolar collapse, (3) inflammation, and (4) septal thickening. In addition, the extent of each histologic feature per field of view was graded from 0 (no visible evidence) to 4 (complete involvement). Scores were calculated as the product of the severity and extent of each feature in a range of 0–16. The cumulative DAD score was calculated as the sum of each score characteristic and ranged from 0 to 64.

Liver histologic assessment was performed using scanned photomicrographs at a magnification of ×400 obtained from ten non-overlapping fields per animal, using Dear's adapted score[5]. Briefly, scores of 0–4 were used to represent hepatocyte lesions: 0, no visible evidence of vacuolization; 1, vacuolization present in >1%–25% of the tissue area; 2,

>25%–50%; 3, 50%–75%; 4, vacuolization present in >75% of the tissue area. Inflammation in liver was quantified by counting the total cells in sinusoids.

Kidney histologic assessment was performed using scanned photomicrographs at a magnification of ×400, obtained from ten non-overlapping fields of view per animal, based on pathology scores developed by a specialist (C.M.T.). Briefly, scores of 0–4 were used to represent tubular cell damage, defined as loss of brush border: 0, no visible evidence; 1, lesion present in >1%–25% of the tissue area; 2, >25%–50%; 3, 50%–75%; 4, lesion present in >75% of the tissue area. ImagePro software (version 4.0; Media Cybernetics, Silver Spring, MD, USA) was used to record the areas occupied by interstitial edema, which were then divided by the total area examined.

Lung, kidney, and liver histologic scores were evaluated by three blinded examiners (N.G.B., J.D.S., C.M.T.).

References

[1] J. D. Silva, G. P. Oliveira, and P. R. M. et al Rocco, "Respiratory and Systemic Effects of LASSBio596 Plus Surfactant in Experimental Acute Respiratory Distress Syndrome," *Cellular Physiology and Biochemistry*, vol. 38, pp. 821–835, 2016, doi: 10.1159/000443037.

[2] M. F. Osuchowski *et al.*, "Minimum quality threshold in pre-clinical sepsis studies (mqtipss): An international expert consensus initiative for improvement of animal modeling in sepsis," *Shock*, vol. 50, no. 4, pp. 377–380, 2018, doi: 10.1097/SHK.0000000000001212.

[3] et al. Gonçalves-de-Albuquerque CF, Medeiros- de-Moraes IM, Oliveira FMdJ, Burth P, Bozza PT, Castro Faria MV, "Omega-9 Oleic Acid Induces Fatty Acid Oxidation and Decreases Organ Dysfunction and Mortality in Experimental Sepsis," *PLoS One*, vol. 11, no. 4, pp. 1–18, 2016, doi: 10.1371/journal.pone.0153607.

[4] T. Kiss *et al.*, "Comparison of different degrees of variability in tidal volume to prevent deterioration of respiratory system elastance in experimental acute lung in fl ammation," *Br J Anaesth*, vol. 116, no. 5, pp. 708–715, 2016, doi: 10.1093/bja/aew093.

[5] J. W. Dear *et al.*, "Sepsis-induced organ failure is mediated by different pathways in the kidney and liver: acute renal failure is dependent on MyD88 but not renal cell apoptosis James," *Kidney Int*, vol. 69, no. 5, pp. 832–836, 2008.

Table S1. PCR Primers

Gene	Forward	Reverse
IL-18	5′-ACA GTG AAG TCG GCC AAA GT-3′	5′-TCT TGG CCC AGG AAC AAT GG-3′
IL-6	5′-TCT CTG GGA AAT CGT GGA A-3′	5′-TCT GCA AGT GCA TCA TCG T-3′
IL-10	5′-ATC CAA GAC AAC ACT ACT ATA-3′	5′-TAA ATA TCC TCA AAG TCC C-3′
KIM-1	5′-GCT AGC ATG CAT CCT CAA GTG GTC-3′	5′-CTC GAG TTA GTC CGT GGC ATA AAG-3′
PD-1	5′-CGT CCC TCA GTC AAG AGG AG-3′	5′-GTC CTT AGA AGT GCC CAA CA-3′
KIM-1	5′-GCT GCT ACT GCT CCT TGT GA-3′	5′-GGA AGG CAA CCA CGC TTA GA-3′
36B4	5′-AAC CCA GTT CTG GAG AAA C-3′	5′-GTT CTG AGC TCC CAC AGT GA-3′

IL, interleukin; PD-1, programmed death 1; KIM-1, kidney injury molecule; 36B4, acidic ribosomal phosphoprotein P0.

Table S2. The Proteins Identified with Significant Intensity

Actg1	actin cytoplasmic 2
AldoA	fructose bisphosphate aldolase A
Anxa5	annexin A5
Anxa6	annexin A6
Atp5a1	ATP synthase subunit alpha, mitochondrial
C3	complement C3
Calr	calreticulin
Col12a1	collagen alpha 1(XII) chain
Col1a1	collagen alpha 1
Cxcl12	stromal cell-derived factor 1
Eef2	elongation factor 2
Fbln2	fibulin 2
Fn1	fibronectin isoform 1
Fn2	fibronectin isoform 2
H2AC12	histone H2A type 1H
Hist1h2ah	histone H2A type 1H
Hsp90b1	endoplasmin
HspA5	78 kDa glucose-regulated protein
Hspg2	basement membrane-specific heparan sulfate proteoglycan core protein
Lamc1	laminin subunit gamma 1
Lox	protein lysine 6 oxidase
Lrif1	ligand-dependent nuclear receptor interacting factor 1
Mdh2	malate dehydrogenase 2
Mif	macrophage migration inhibitory factor
Myh9	myosin 9
Myl12a	myosin light chain 12A
Myl6	myosin light polypeptide 6
Nid 1	nidogen 1
Pdia3	protein disulfide isomerase A3
Plac8	placenta specific 8
Rps5	ribosomal protein S5
S100A4	S100 calcium-binding protein A4
Thbs1	thrombospondin 1
Tnc	tenascin
Tpm1	tropomyosin alpha-1 chain
Vcp	transitional endoplasmic reticulum ATPase

Table S3. WikiPathways: Pathway Enrichment Analysis of Statistically Abundant Proteins

Source	Regulation	Term	*P* value	Adjusted *P* value	Odds Ratio	Combined Score	Genes
BM/ADxL	Up	IL-9 signaling pathway WP10	0.003596	0.004642964	434.2174	2443.769793	VCP
BM/ADxL	Up	Prostaglandin synthesis and regulation WP374	0.004643	0.004642964	332.7833	1787.845974	ANXA5
BM/ADxL	Down	Inflammatory response pathway WP458	0.007478	0.029119416	172.1207	842.6622961	THBS1
BM/ADxL	Down	Striated muscle contraction WP216	0.0112	0.029119416	113.358	509.1809645	TPM1
BM/ADxL	Down	TGF-β signaling pathway WP113	0.012934	0.029119416	97.76471	425.0726939	THBS1
BM/ADxL	Down	p53 signaling WP2902	0.01664	0.029119416	75.48864	309.1988807	THBS1
BM/ADxL	Down	Calcium regulation in the cardiac cell WP553	0.036217	0.050703935	33.98801	112.7798428	CALR
BM/ADxL	Down	Focal adhesion WP85	0.045406	0.052974231	26.91712	83.23043532	THBS1
BM/ADxL	Down	Focal adhesion-PI3K-Akt-mTOR-signaling pathway WP2841	0.078425	0.078425076	15.22601	38.7594974	THBS1
AD/BMxL	Up	Striated muscle contraction WP216	0.022278	0.07086001	50.36869	191.6095305	TPM1
AD/BMxL	Up	Glycolysis and gluconeogenesis WP157	0.025215	0.07086001	44.31111	163.0792417	ALDOA
AD/BMxL	Up	Oxidative phosphorylation WP1248	0.030578	0.07086001	36.30055	126.5975833	ATP5A1
AD/BMxL	Up	Primary focal segmental glomerulosclerosis FSGS WP2573	0.03543	0.07086001	31.17214	104.1210777	MYH9
AD/BMxL	Up	Calcium regulation in the cardiac cell WP553	0.071131	0.087693392	15.10198	39.91798491	CALR
AD/BMxL	Up	MAPK signaling pathway WP493	0.076732	0.087693392	13.94655	35.80694267	HSPA5
AD/BMxL	Up	Focal adhesion WP85	0.088762	0.088761986	11.96014	28.96504081	MYL6
AD/BMxL	Down	Inflammatory response pathway WP458	0.007478	0.023180083	172.1207	842.6622961	FN1
AD/BMxL	Down	Prostaglandin synthesis and regulation WP374	0.007727	0.023180083	166.375	809.0939625	ANXA5
AD/BMxL	Down	Regulation of actin cytoskeleton WP523	0.03743	0.055932453	32.8543	107.9354587	FN1
AD/BMxL	Down	Focal adhesion WP85	0.045406	0.055932453	26.91712	83.23043532	FN1
AD/BMxL	Down	Chemokine signaling pathway WP2292	0.04661	0.055932453	26.19841	80.32255388	CXCL12
AD/BMxL	Down	Focal adhesion-PI3K-Akt-mTOR-signaling pathway WP2841	0.078425	0.078425076	15.22601	38.7594974	FN1
L/BMxAD	Up	Inflammatory response pathway WP458	2.17E-05	1.52E-04	475.4048	5105.230681	COL1A1;THBS1
L/BMxAD	Up	Focal adhesion WP85	8.36E-04	0.002924434	72.17486	511.5333734	COL1A1;THBS1
L/BMxAD	Up	Osteoblast WP238	0.002498	0.004432993	555.1667	3326.770642	COL1A1

L/BMxAD	Up	Focal adhesion-PI3K-Akt-mTOR-signaling pathway WP2841	0.002533	0.004432993	40.73085	243.5010775	COL1A1;THBS1
L/BMxAD	Up	Dysregulated miRNA targeting in insulin/PI3K-AKT signaling WP3855	0.006484	0.009077165	199.7	1006.181577	COL1A1
L/BMxAD	Up	TGF-β signaling pathway WP113	0.012934	0.015089367	97.76471	425.0726939	THBS1
L/BMxAD	Up	p53 signaling WP2902	0.01664	0.016639666	75.48864	309.1988807	THBS1

AD, adipose tissue; BM, bone marrow; L, lung tissue.

Figure S1. Cytokines in liver parenchyma

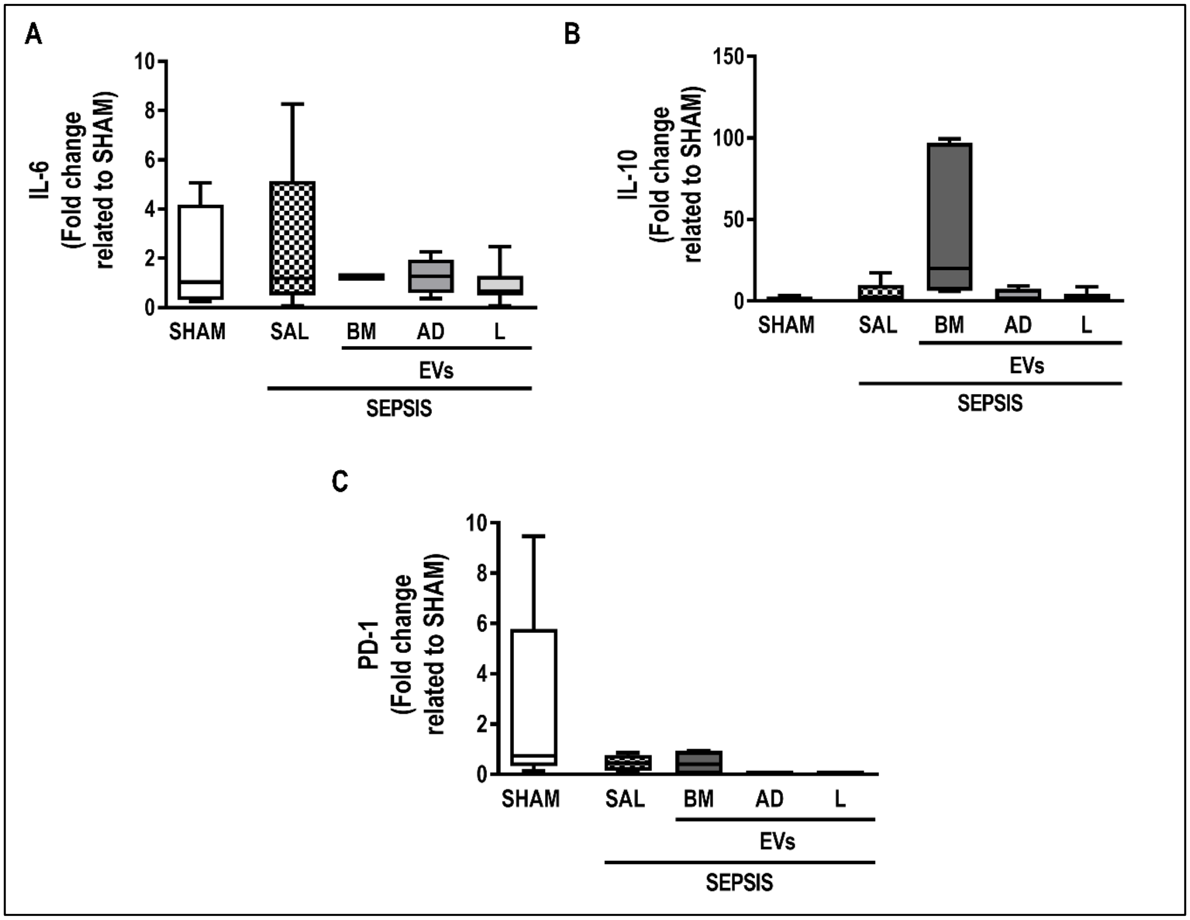

Figure S1. (A) interleukin (IL)-6 levels; (B) IL-10; and (C) programmed cell death protein (PD)-1 levels quantified by PCR.
